# NEDD: a network embedding based method for predicting drug-disease associations

**DOI:** 10.1186/s12859-020-03682-4

**Published:** 2020-09-17

**Authors:** Renyi Zhou, Zhangli Lu, Huimin Luo, Ju Xiang, Min Zeng, Min Li

**Affiliations:** 1 Hunan Provincial Key Lab on Bioinformatics, School of Computer Science and Engineering, Central South University, Changsha, China; 2 School of Computer and Information Engineering, Henan University, Kaifeng, 475001 China; 3 Neuroscience Research Center & School of Basic Medical Sciences, Changsha Medical University, Changsha, China

**Keywords:** Drug repositioning, Heterogeneous network, Network embedding, Meta path

## Abstract

**Background:**

Drug discovery is known for the large amount of money and time it consumes and the high risk it takes. Drug repositioning has, therefore, become a popular approach to save time and cost by finding novel indications for approved drugs. In order to distinguish these novel indications accurately in a great many of latent associations between drugs and diseases, it is necessary to exploit abundant heterogeneous information about drugs and diseases.

**Results:**

In this article, we propose a meta-path-based computational method called NEDD to predict novel associations between drugs and diseases using heterogeneous information. First, we construct a heterogeneous network as an undirected graph by integrating drug-drug similarity, disease-disease similarity, and known drug-disease associations. NEDD uses meta paths of different lengths to explicitly capture the indirect relationships, or high order proximity, within drugs and diseases, by which the low dimensional representation vectors of drugs and diseases are obtained. NEDD then uses a random forest classifier to predict novel associations between drugs and diseases.

**Conclusions:**

The experiments on a gold standard dataset which contains 1933 validated drug–disease associations show that NEDD produces superior prediction results compared with the state-of-the-art approaches.

## Background

Drug discovery is known for a large amount of money and time it consumes and the high risk it takes [1]. The investments grow continuously in recent years, but the total number of approved drugs remains constant [2]. Therefore, drug repositioning has become a popular approach to save cost by finding novel indications for approved drugs. Since these commercialized drugs have passed various clinical tests, it would save tremendous effort if we could reuse them directly. As reported, relaunching a repositioned drug can save about 80% of the cost compared with launching a reformulation of an existing drug [3].

The goal of drug repositioning is to find potential new target diseases for an existing drug and apply the newly identified drug to the treatment of diseases other than the drug’s originally intended ones [4]. Historically, the discovery of finding new indications for existing drugs is mostly the result of a better understanding of a drug [5] or serendipity [6]. Then as the omics data accumulate, new bioinformatics methods emerge and play an increasingly important role. The newly proposed methods generally can be categorized as ‘drug based’ or ‘disease based’ [7]. For instance, IDMap [8] is a drug-based method and it mainly focuses on exploring the chemical structure information of drugs. Later, with the growth of drug-related data and the initiative of open data, recent studies pay more attention to integrating heterogeneous information. For example, Gottlieb et al. proposed PREDICT [9], a method that integrates various drug-drug similarity and disease-disease similarity from different sources.

These computational repositioning approaches can be roughly divided into three types: machine learning methods, text mining methods, and network-based methods [5, 10].

The aforementioned method PREDICT is an example of machine learning methods. The authors used similarities as features and applied a logistic regression classifier to predict novel indications for drugs. Moreover, Napolitano et al. proposed an approach which used a combination of drug-related data to train a multi-class SVM (Support Vector Machine) classifier to identify latent drug-disease associations [11]. Besides, there are also researches comparing traditional machine learning methods and deep learning methods [12, 13].

Electronic health records (EHR) of the patients and other literature contains a vast amount of information about drugs and diseases that can be explored using the text mining technique. For instance, Zhu et al. explored pharmacogenomics studies and modelled FDA-approved breast cancer drugs by using Semantic Web notions which support automated semantic inference [14]. Chen et al. integrated and annotated data from public datasets and developed a statistical model called Semantic Link Association Prediction (SLAP) to assess drug–target associations based on semantic links [15].

Network-based methods have been wildly used for computational drug repositioning. Martínez V et al. proposed DrugNet [16], which is based on a heterogeneous network prioritization approach that can utilize heterogeneous information. Luo, Y et al. developed a pipeline called DTINet [17]. It originally aims to find interactions between drugs and targets, but can also be applied in drug repositioning. DTINet uses a matrix completion method to calculate the low dimensional feature vectors which capture the topological properties of nodes in the network and uses these features to predict novel associations. Luo, H et al. proposed MBiRW [18], which used the Bi-Random Walk algorithm to predict potential novel indications of a drug. However, current network-based methods often show a certain preference for drugs that have more known drug-disease associations. Therefore, they are not good at finding novel indications for drugs that are less explored or new drugs.

In this work, we propose NEDD, a network embedding based method for predicting novel interactions between drugs and diseases using heterogeneous information. NEDD tries to solve the above problems by adopting the concept of inductive learning and meta path. The results of experiments show that NEDD outperforms other methods in drug repositioning.

## Methods

In this section, we will introduce our method NEDD. Generally, the whole procedure of NEDD consists of three steps. First, based on the heterogeneous information related to drugs and diseases, an undirected graph with weighted edges is constructed. Second, we train a meta-path-based representation learning model to learn the embedding vectors of each entity. Last, using the vectors learned, we train a classifier to identify potential associations between drugs and diseases.

### Construction of the drug-disease network

We construct the network as an undirected graph that consists of two node types (drug node *n*_*dr*_ and disease node *n*_*di*_) and three edge types (drug-disease edge *r*_*dr-di*_, drug-drug edge *r*_*dr-dr*_, and disease-disease edge *r*_*di-di*_). An edge of *r*_*dr-di*_ represents the association between a drug and a disease. An edge of *r*_*dr-dr*_ represents the connection between two drugs which have a high similarity and an edge of *r*_*di-di*_ denotes the connection between two diseases which have a high similarity.

**Fig. 1:**
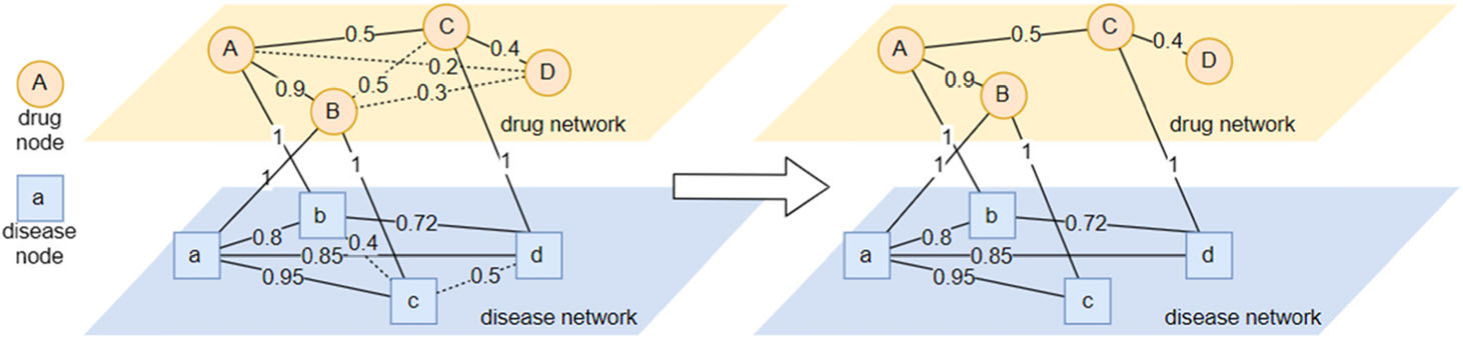
Network Construction. A demo graph constructed by integrating drug similarity network and disease similarity network. Dotted lines, like Edge_bc_, represent removed edges whose weights under thresholds. Though weights of Edge_AC_ is under the threshold, this edge is not removed because firstly all similarity edges of Node_*c*_ are below the threshold and secondly it is the edge with the most weight among them. And it is the same with *Edge*_*CD*_

After calculating all the similarities among drugs and that among diseases, we filter the edges with a certain threshold. In particular, the threshold of drug similarity is 0.8 and the threshold of disease similarity is 0.7. The thresholds are determined by experiments. Each similarity edge below its threshold is removed unless it has the greatest weight (similarity) among other homogeneous edges for a drug or a disease and removing this edge may cause one node to be isolated. The insight of filtering is that drug pairs with low similarity have an insignificant probability in indicating common diseases while drug pairs with high similarity have a strong probability to indicate common diseases [18], and the same is also true for disease pairs. This step is illustrated in Fig. 1.

Though here we only make use of three types of relationships within drugs and diseases, other types of information, like disease-target interactions and so on, can be used to expand the graph.

### Network embedding

Most existing network representation learning methods can be summarized into two steps: proximity matrix construction and dimension reduction [19]. NEDD uses random walk to perform the first step. One of the strengths of random walk is that it is efficient in both time and space. In the next step, NEDD adopts a neural-network-based method, HIN2vec [20], to learn network embedding vectors.

The key concept of the first step is the meta path. Given a drug-disease network *G*(*V,E*), the node type set *T*_*V*_ and the edge type set *T*_*E*_, a meta path can be defined as a triplet (*n*_*h*_, *n*_*t*_, *m*), where n_h_, n_t_∈*T*_*V*_ are the head node type and the end node type, and m is a sequence of edge types *r*_1_ → *r*_2_ → … → *r*_*l*_ (r_1_, r_2_, …, r_l_ ∈*T*_*E*_) which indicates a composite relation between the two node types. For example, a meta path (*n*_*di*_, *n*_*di*_, *r*_*dr-di*_→*r*_*dr-di*_) indicates the relationship that two diseases share the same treatment, while a meta path (*n*_*dr*_*, n*_*dr*_, *r*_*dr-di*_*→r*_*di-di*_*→r*_*dr-di*_) describes the relationship of two drugs that could treat two similar diseases.

NEDD first uses a random walk algorithm to generate long possible paths in the graph. In this process, node numbers and node types are recorded. Then, these paths are cut into shorter paths with lengths from 1 to W. These shorter paths represent the network proximity between two nodes of the 1^*st*^ to the *W*^*th*^ order. In our experiments, W is set to 6. Besides, negative sampling [21] is used to generate negative data entries.

Then, NEDD adopts HIN2vec [20] to learn network embedding vectors. HIN2vec is a representation learning method. It assigns a low dimensional embedding vector for each entity in the graph, including drug nodes and disease nodes, and each meta path. It trains a neural network classifier with one hidden layer to identify if two nodes have a certain relationship and take weights of the hidden layer as embedding vectors like word2vec [21]. The prediction which node *v*_*i*_ and node *v*_*j*_ have an association that matches a particular meta path *R* is given as below:

$$ P\left(R|{v}_i,{v}_j\right)= sigmoid\left(\sum {e}_{v_i}\odot {e}_{v_j}\odot {f}_{01}\left({e}_R\right)\ \right) $$

where ${e}_{v_i},{e}_{v_j},{e}_R\in {R}^D$ is the embedding vector of the nodes *v*_*i*_, *v*_*j*_, and the meta path *R*, *f*_01_ is a regularization function that regularizes values in *e*_*R*_ within 0 and 1, and ⊙ is the element-wise product. HIN2vec uses cross-entropy loss to measure the prediction error and uses stochastic gradient descent to update embedding vectors.

**Fig. 2:**
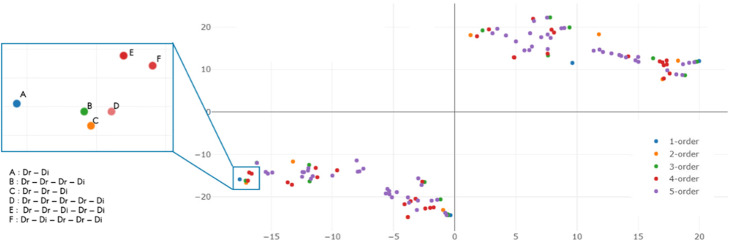
Visualization of meta path embedding vectors. Visualization of meta path embedding vectors using T-SNE [22]. The visualization result is very intuitive. Since the graph we created is an undirected one, the embeddings of paths are naturally symmetric. The lower-left group is meta paths which start from drug nodes and the upper-right group represent meta paths which start from disease nodes. Each point represents a meta path vector and different color represents different order of the relationships. For instance, point C on the left is a meta path of “Drug-Drug-Disease”, which represents the second order relationship that a drug might cure a disease that a similar drug can treat

A visualization using T-SNE [22] of meta path embedding vectors is shown in Fig. 2.

### Using embedding vectors to predict novel associations

In this step, we use a random forest classifier to make final predictions. We apply the element-wise product to aggregate a drug node embedding vector and a disease node embedding vector together as the input of the random forest classifier. The output of the random forest classifier is the predicted probability that a drug and a disease have associations:

$$ P\left( dur{g}_i\  treats\ diseas{e}_j\right)= random\_ forest\_ classifier\left({e}_{dru{g}_i}\odot {e}_{diseas{e}_j}\right) $$

We use scikit-learn [23] to implement the random forest classifier. While optimizing the parameters of the random forest, we evaluated the forest on the same training set and validation set. Because random forests are less likely to overfit, we started with a large classifier and gradually cut down the scale. In the end, we set the max depth of trees to 25, the number of estimators to 300, the minimum number of samples to split to 2, and use the Gini coefficient as the criterion.

## Results

In this section, we evaluate the performance of NEDD on the gold standard dataset. First, we introduce the evaluation metrics. Then, by performing these measurements, we compare NEDD with five other methods: MBiRW [18], DTINet [17], HGBI [24], NBI [25], and JUST [26].

### Dataset

In the dataset, drug-disease associations are collected from multiple data sources. This gold standard dataset which has been used in reference [9] includes 593 drugs from DrugBank [27], 313 diseases from the Online Mendelian Inheritance in Man (OMIM) [28] and 1933 validated drug–disease associations.

The drug similarity data is calculated by the Chemical Development Kit (CDK) [29] based on SMILES [30] chemical structures and the disease similarity data is obtained from MimMiner [31] which is based on disease phenotype similarity using text mining analysis of their medical descriptions information in the OMIM database.

### Evaluation metrics

In order to evaluate the ability of NEDD in finding new possible target diseases of a specific drug, we conduct 10-fold cross-validation and perform the top-ranked candidate disease analysis.

In 10-fold cross-validation, all 1933 known drug-disease associations in gold standard datasets are randomly divided into 10 partitions with each roughly equal in size. Then, 1 of 10 partitions in turn serves as the test set, while the remaining as the training set. After the whole process, each possible association is given a score representing the confidence of the association. Then these associations are sorted in descending order according to their score. Next, for each ranking threshold, true positive rate (TPR), which measures the proportion of known associations that are correctly identified, and false positive rate (FPR), which measures the proportion of unverified associations that are predicted as real associations, are calculated based on the ranking results. By changing settings of the threshold, we can get various pairs of TPR and FPR. Based on these pairs, the receiver operating characteristic curve (ROC curve) can be drawn with FPR as the x-axis and TPR as the y-axis. The area under ROC (AUC) is then calculated to measure the performance.

Besides, as top ranked predicting results may be important in practice, we also test our method in terms of top ranked results. We count the number of true drug-disease associations proved by other sources, e.g. other datasets or literatures to evaluate NEDD and other methods.

### Comparison with other methods

NEDD is compared with five state-of-the-art methods: MBiRW [18], DTINet [17], HGBI [24], NBI [25], and JUST [26]. These five methods are all computational methods which are based on network and can utilize the heterogeneous network of drugs and diseases. NBI is a method based on a two-state diffusion model in a bipartite graph. HGBI is a method based on the guilt-by-association principle and an intuitive interpretation of information flow on a heterogeneous graph. MBiRW and DTINet have been introduced in the above. MBiRW is based on random walk and DTINet is based on matrix completion. Moreover, similar to NEDD, DTINet also adopts the concept of inductive learning. HGBI and NBI are also originally developed for drug-target association prediction but they have also been used in drug-disease association prediction [32]. JUST improves the random walk sampling method on the heterogeneous network which avoids using meta paths and use Skip-Gram model [21] to learn network embedding vectors. In the study, the parameter *α* is set to 0.3, and *l*, *r* to 2 for MBiRW according to the default parameter setting in [18]. The parameter *α* of HGBI is set to 0.4 as suggested in [24]. We change the lengths of vectors to 100 in DTINet, and we use the default settings for other parameters. Besides, we use additional information to train DTINet model because the method mainly focuses on integrating various information. The additional information includes drug-drug interactions and drug-protein interactions collected from DrugBank [27], associations between drugs and side-effects from SIDER [33], and disease-gene associations from CTD [34]. For JUST, we set *α* to 0.4, *m* to 2 as suggested in the original paper [26]. The length of the embedding vectors is set to 128 and the window size of the Skip-Gram model is set to 10.

**Fig. 3:**
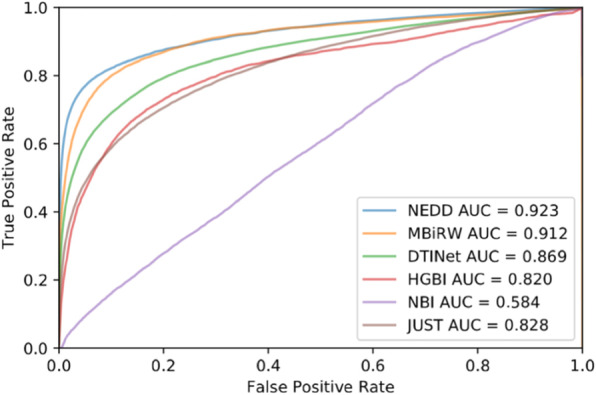
Results of ten-fold cross-validation. AUC and ROC curve of ten-fold cross-validation

Through repeating the 10-fold cross-validation experiment specified in the above 1 hundred times with different random seeds, we calculate average AUC to estimate the performance of each method. In the 10-fold cross-validation test, the experiment results show that NEDD outperforms the other methods. NEDD achieves the AUC value of 0.923, while MBiRW, DTINet, HGBI, NBI, and JUST obtain inferior results of 0.912, 0.869, 0.820, 0.584 and 0.828, respectively. The results are illustrated in Fig. 3.

### Case study

**Fig. 4:**
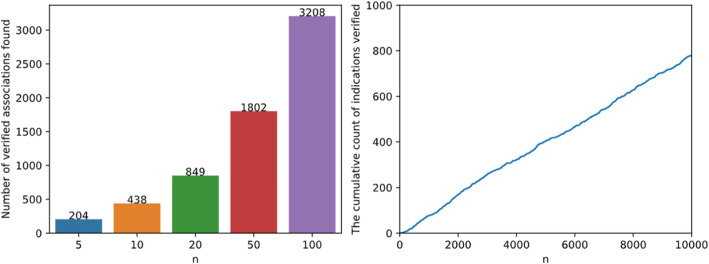
Result of top n test. Number of verified novel drug–disease associations found by NEDD. On the left is the sum of verified associations which rank in the top *n* results for each drug. On the right is the number of verified associations found in the top *n* results of all

After confirming the ability of NEDD to predict potential drug-disease associations based on 10-fold cross-validation, we further conduct a case study to search evidences in other sources. In this step, all known associations in the gold standard dataset are used as the training set, and then the possible associations are ranked according to NEDD’s prediction. Next, top-ranked predictions for each drug are verified based on CTD [34]. The top-N results of NEDD are summarized in Fig. 4. In this step, it shows that 204 novel indications in the top 5 predictions are verified by CTD; 438 novel indications are verified in the top 10 and 849 novel indications are verified in the top 20.

Fifteen drug-disease associations with the highest prediction scores of all are listed in Table 1. None of these associations are verified by CTD. So, we conduct a further study to find supporting evidences by literature searching. The result shows that 7 out of 15 predictions are verified by literature. In predictions ranked 1 and 6, drugs are related to corresponding diseases, but may target another subtype of the diseases. Predictions ranked 2, 3, 5, 7, 11, and 12 have not been adequately investigated. Some details are provided below.

**Table 1:** Associations with the highest prediction scores

Rank	Drug		Disease		References
	ID (DrugBank)	Name	ID (OMIM)	Name	
**1**	DB01202	Levetiracetam	208,700	ATAXIA WITH MYOCLONIC EPILEPSY AND PRESENILE DEMENTIA	[35]
**2**	DB01181	Ifosfamide	267,730	RETICULUM CELL SARCOMA	–
**3**	DB00937	Diethylpropion	303,110	CHOROIDEREMIA, DEAFNESS, AND MENTAL RETARDATION	–
**4**	DB00584	Enalapril	161,900	RENAL FAILURE, PROGRESSIVE, WITH HYPERTENSION; RFH1	[36]
**5**	DB00444	Teniposide	276,300	MISMATCH REPAIR CANCER SYNDROME; MMRCS	–
**6**	DB01070	Dihydrotachysterol	277,440	VITAMIN D-DEPENDENT RICKETS, TYPE 2A; VDDR2A	[37]
**7**	DB00176	Fluvoxamine	131,300	CAMURATI-ENGELMANN DISEASE; CAEND	–
**8**	DB00710	Ibandronate	167,320	INCLUSION BODY MYOPATHY WITH EARLY-ONSET PAGET DISEASE WITH OR WITHOUT FRONTOTEMPORAL DEMENTIA 1; IBMPFD1	[38]
**9**	DB00710	Ibandronate	602,080	PAGET DISEASE OF BONE 2, EARLY-ONSET; PDB2	[38]
**10**	DB00282	Pamidronic acid	602,080	PAGET DISEASE OF BONE 2, EARLY-ONSET; PDB2	[39]
**11**	DB01551	Dihydrocodeine	147,530	INSENSITIVITY TO PAIN WITH HYPERPLASTIC MYELINOPATHY	–
**12**	DB00500	Tolmetin	147,530	INSENSITIVITY TO PAIN WITH HYPERPLASTIC MYELINOPATHY	–
**13**	DB00136	Calcitriol	241,519	HYPOPHOSPHATEMIA, RENAL, WITH INTRACEREBRAL CALCIFICATIONS	[40]
**14**	DB00214	Torasemide	256,370	NEPHROTIC SYNDROME, TYPE 4; NPHS4	[41]
**15**	DB01120	Gliclazide	600,496	MATURITY-ONSET DIABETES OF THE YOUNG, TYPE 3; MODY3	[42]

Levetiracetam can treat epilepsy but one of its side-effects is ataxia [35]. So, it may not be helpful in treating ataxia with myoclonic epilepsy and presenile dementia. Ifosfamide is predicted to treat reticulum cell sarcomaand it has been used in treating soft tissue sarcoma [43]. Enalapril is an orally-active antihypertensive agent that can suppress the renin-angiotensin-aldosterone system to lower blood pressure. NEDD predicts Enalapril can treat renal failure, which has been tested in [36]. Teniposide is used for the treatment of refractory acute lymphoblastic leukaemia. The prediction result suggests that it may be applied in the treatment of mismatch repair cancer syndrome (MMRCS) as well. Dihydrotachysterol is predicted to treat vitamin D-dependent rickets, type 2a, which has been studied in [37]. NEDD suggests using Ibandronate in the treatment of inclusion body myopathy with early-onset Paget disease with or without frontotemporal dementia 1 and the treatment of Paget disease of bone 2, early-onset. And Ibandronate has long been used in treating Paget disease [38]. Pamidronic acid is used to prevent bone loss and to strengthen the bone in Paget disease [39], which verified the prediction of our method. Calcitriol, an active metabolite of vitamin D is predicted to treat renal hypophosphatemia with intracerebral calcifications. In real life, it is used in the treatment of hypophosphatemia [40]. Despite concerns that the use of calcitriol may contribute to vascular calcification, there is no clear evidence [44]. Torasemide is a high-ceiling loop diuretic [45]. It is predicted to treat nephrotic syndrome, type 4; this prediction is verified in [41]. For Gliclazide, the prediction to treat maturity-onset diabetes of the young of type 3 has been tested in clinical trials [42].

### Parameter sensitivity

In this section, we investigate the parameter sensitivity. We change the thresholds for drug similarities and disease similarities and the window size to see how these parameters affect the result. We conduct 10-fold cross-validation five times for each parameter setting and evaluate the performance using AUC values. In each experiment, we only change one corresponding parameter and set the others as default—i.e. 0.7 for disease similarity threshold, 0.8 for drug similarity threshold, and 6 for window size.

**Fig. 5:**
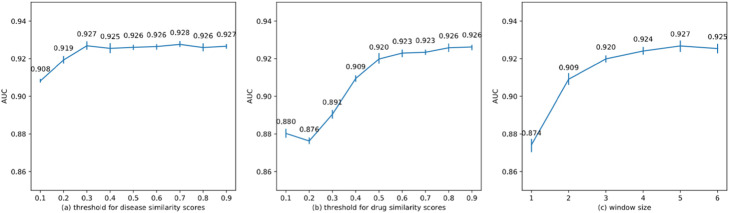
Result of parameter sensitivity test. AUC of ten-fold cross-validation on different parameter settings

The results are illustrated in Fig. 5. From Fig. 5, we can recognize a similar pattern. The performance rises initially when the values of the corresponding parameters rise. However, after a certain point, NEDD becomes insensitive towards that parameter. For window size, this is because most of the useful information is already encoded in the embedding vectors. For thresholds of similarity scores, this is because most of the noise has already been ruled out at the beginning when we raise the thresholds.

### Model robustness

In this section, we investigate model robustness over different similarity measures.

We evaluate NEDD’s performance using three different disease similarity measures, i.e. MimMiner [31] which is in the golden test dataset and utilizes disease phenotype information, NetSim [46] which employs the protein interaction network, and RADAR [47] which we used to get similarity scores based on pathways. Since we use the disease similarity scores provided by the authors and they used other types of ID for disease and some IDs do not have any mapping information, the experiments are done on a subset of the original dataset, which consists of 196 diseases, 593 drugs, and 1052 drug-disease associations.

We also evaluate NEDD’s performance using three different drug similarity measures which utilize the information from the chemical structure, the corresponding side effects of each drug and the drug-related genes respectively. The three different types of drug similarity are calculated according to [9].

**Fig. 6:**
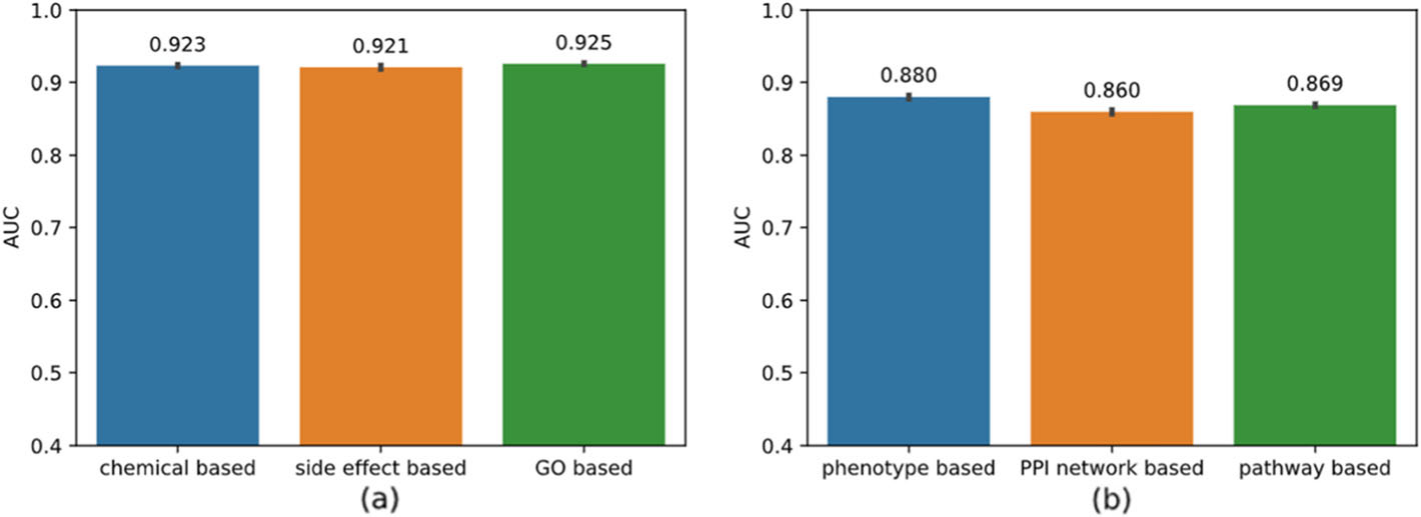
Result of model robustness test. **a** AUC of ten-fold cross-validation tests over different drug similarity measures; **b** AUC of ten-fold cross-validation tests over different disease similarity measures on a subset of the original dataset

We repeat 10-fold cross-validation 10 times on each type of similarity scores. The results are illustrated in Fig. 6, in which NEDD produces similar AUC over different disease similarity measures.

## Discussion

We think that the superior performance of NEDD in finding indications for new drugs stems from two aspects: inductive learning and meta path. Inductive learning methods can be applied to entities not seen at train time. Compared with MBiRW, HGBI and NBI, DTINet and NEDD, which used inductive methods, yields relatively higher AUC score in the test. And with the concept of meta path, NEDD is able to explicitly capture high order proximities. This is especially important in tasks like drug repositioning where many latent links between drugs and diseases are unknown. If the associations between two nodes are missing, their first-order proximity is zero, so it is essential to exploit high order information.

Besides, NEDD can be easily adopted in larger datasets with more types of biological entities such as target, gene, side-effect, etc.

However, the limitations of NEDD should also be acknowledged. First, in order to use various information like drug-target interactions, the maximum length of the meta path should be increased, which might significantly increase the computational cost. Second, because the trained embeddings are not specifically fine-tuned for association prediction between drugs and diseases, the difficulty in training the vectors is increased when adding more information to the network.

## Conclusion

In this work, we present NEDD, a new computational approach for drug repositioning. NEDD uses a meta-path-based representation method to inductively learn node embedding vectors of drugs and diseases on a given graph. The graph is constructed by integrating heterogeneous biological information related to drugs and diseases. After learning the network embedding vectors, a random forest classifier is trained to predict the probabilities of drugs and diseases being associated.

NEDD shows competitive results in the 10-fold cross-validation test. The case study of NEDD gives fair results that remain to be further explored. In summary, the results prove that NEDD is practical in drug repositioning tasks toward existing drugs.

## Data Availability

The used dataset can be downloaded from bioinformatics.csu.edu.cn/resources/softs/DrugRepositioning/n_Web/NEDD.html.

## Abbreviations

SVM: Support vector machine
HER: Electronic health records
OMIM: Online Mendelian Inheritance in Man
CDK: Chemical Development Kit
TPR: True positive rate
FPR: False positive rate
AUC: Area under curve
ROC: Receiver operating characteristic
